# Medical Society of the County of Erie

**Published:** 1908-08

**Authors:** Franklin C. Gram

**Affiliations:** Secretary


### SOCIETY PROCEEDINGS.

### Medical Society of the County of Erie.

Reported by FRANKLIN C. GRAM, M. D., Secretary.

THE regular quarterly meeting of the Medical Society of the County of Erie was held at 4 p. m., June 15, 1908, in the rooms of the Society of Natural Sciences, Buffalo Library Building, the president Dr. Edward Clark, being in the chair.

The secretary read the minutes of the quarterly meeting held April 20, 1908, which were received. The amendments to the by-laws which were offered at that meeting were then considered seriatim.

Amendment No. 2 was changed to read "when at least twenty members are present." The other by-laws were each adopted as read. On motion of Dr. Wall, all the minutes and the proposed by-laws were then approved and adopted.

The secretary then read the minutes of the meeting of the Council held June 10, 1908, all of which were approved as read.

The secertary read the resignation of Dr. Harriet E. Sheldon, which was accepted.

In compliance with the direction of the council, the treasurer, Dr. Lytle, then read the names of all those who were in arrears. All were suspended by action of the by-laws. The treasurer explained the careful manner in which he had dealt with each individual before referring them to the council which again considered each name before it was placed on this list.

On motion of Dr. Wall, the names of all those who were read and the entire subject matter of suspension and reinstatement was referred to the council with power. The treasurer then read the names of those who had not paid their dues up to May 1, 1908.

Dr. T. H. McKee, Chairman of the Committee on Membership, then read the list of applicants and recommended their election as members of this society. Each name was considered individually and all were elected.

The names of those elected are as follows:

Henry J. Siedler, 1312 Fillmore Ave., Buffalo; David Cohn, 141 N. Division Street, Buffalo; Floyd Richardson, East Aurora; Albert W. Phelps, East Aurora; Anna M. Reinstein, 521 Broadway, Buffalo; Descum C. McKenney, 1250 Main Street, Buffalo; Harry A. Wood, 2200 Main Street, Buffalo; James A. Gardner,

403 Franklin Street, Buffalo; Frank M. Sweetland, Angola; Richard Hirsch, 1555 Genesee Street, Buffalo; George B. Dandy, 232 Norwood Avenue, Buffalo; Albert E. Persons, 27 W. Tupper Street, Buffalo; John C. Kamp, 1019 Grant Street, Buffalo.

The censors, through Dr. Grant, reported progress.

By special action taken at the annual meeting, the nominations for 1909 were directed to be made at this meeting. The president called for nominations for each of the offices to be filled, with the following result:

For president, Charles A. Wall; for first vice-president, Grover W. Wende; for second vice-president, Bernard Cohen; for secretary, Franklin C. Gram; for treasurer, Albert T. Lytle; for censors, Henry R. Hopkins, chairman, and the following members: DeLancey Rochester, Francis E. Fronczak, Walter D. Greene and John H. Grant.

For chairman of committee on legislation—F. Park Lewis; for chairman of committee on public health—Ernest Wende; for chairman of committee on membership—Thomas H. McKee; for delegates to the Medical Society of the State of New York for two years—Charles A. Wall, Arthur G. Bennett, Eli H. Long, Edward Clark, John H. Grant, J. D. Bonnar, Bernard Cohen, J. F. Rice and T. H. McKee.

For delegates to the Eighth District Branch,—James Stoddart, J. D. Bonnar, Albert T. Lytle, William H. Thornton, J. W. Grosvenor, Edward Blaauw, William C. Krauss, Edward Clark, B. P. Hoyer, Frank M. Sweetland, H. G. Hopkins, William Irving Thornton, Bentley S. Bourne, F. H. Stanbro and Albert W. Phelps.

Dr. F. Park Lewis then offered the following preamble and resolutions:

"WHEREAS, Ophthalmia Neonatorum, which is well-known as a preventable and controllable infection of the eyes, is still producing a large amount of blindness, notwithstanding the greater care exercised by individual obstetricians, and

WHEREAS, The American Medical Association has recommended that an organised movement be conducted throughout the various state and county medical societies for the prevention and control of this disease,

*Resolved,* That the Medical Society of the County of Erie approves the efforts that are being made in this direction, and

*Resolved,* That a committee, one member of which shall be the Health Physician of the city, be appointed by the president of this society, and whose duty shall be to put the recommendations of the Committee on Ophthalmia Neonatorum of the American Medical Association into effect in the County of Erie as far as may seem to be practicable."

Dr. Wall moved that the resolutions be so amended as to make this committee consist of Dr. F. Park Lewis as chairman, with the Health Commissioner of this city, and one other member and whom they shall recommend to the council. Dr. Lewis accepted the amendment and the motion, as amended, was then adopted.

The president stated that formerly, when a member died, special meetings of the society would be called to take appropriate action. Since the society had become so large, this custom had fallen somewhat into disuse. He, therefore, with the consent of the society, appointed a committee on necrology whose duties it should be to prepare suitable resolutions upon the death of all members who died during the year, and present them to the society at the annual meeting. He named, as such committee, Dr. J. W. Grosvenor, Dr. DeLancey Rochester and Dr. William T. Getman.

Dr. Bonnar stated that the question of quarantine in cases of contagious disease often became a hardship to the afflicted families and thought the common council and the mayor should be memorialised on the matter and the question of providing suitable hospital accommodations by the city. He, therefore, offered the following resolution:

"That the Medical Society of the County of Erie memorialise the common council and the mayor on the necessity of providing a municipal hospital for contagious diseases."

Dr. Wall offered an amendment to the resolution empowering the president of this society to appoint a committee of fifteen to take up this matter with the common council and the mayor. The motion, as amended, was then adopted.

The president later appointed the following-named members of this committee: J. D. Bonnar, chairman, and Grover W. Wende, Charles A. Wall, F. C. Gram, Ernest Wende, A. H. Briggs, P. W. Van Peyma, W. D. Greene, H. R. Hopkins, H. G. Matzinger, F. E. Fronczak, W. C. Callahan, DeLancey Rochester, Charles G. Stockton and D. W. Sherman.

The scientific session was called to order by the president at 8.30 p.m., when five minute talks were given as follows:

H. C. Matzinger, "Mental Changes Associated with Prolonged Visceral Diseases;" Dewitt G. Wilcox, "Tubercular Peritonitis;" S. A. Dunham, "A Case of Paranoia;" Marshall Clinton, "Intestinal Obstruction;" F. Park Lewis, "Ophthalmia Neonatorum;" E. C. Mann, "The Laity and Disease;" J. A. Gardner, "A Plea for Support of the Buffalo Branch of the Society for Sanitary and Moral Prophylaxis."

At the conclusion of these talks, every subject presented was discussed. The president then thanked all those who had favored the society with these scientific talks, and the meeting adjourned.

